# Malignant Fibrous Tumour of the Humerus, Necessitating Removal of the Os Humeri at the Shoulder Joint

**Published:** 1862-06

**Authors:** Charles Winne

**Affiliations:** Buffalo


### ART. VII.—*Malignant Fibrous Tumour of the Humerus, necessitating removal of the Os Humeri at the Shoulder Joint; by* CHARLES WINNE, M. D.

In the early part of April, 1862, Mrs. Rogers was admitted into the Hospital of the Sisters of Charity. She was about thirty-five years of age, had several children. She had suffered but little from sickness during her life, and had been active and energetic. A year and a half ago she observed a small, hard tumour upon the exterior of the outer condyle of the right os humeri, which increased gradually for a year. Within the past six months it has enlarged rapidly, attaining a size of from five to six inches in diameter. In January the surface became red, painful, and suppuration ensued from several points, from which the skin sloughed, leaving a jagged, ulcerated surface; from these several chasms fungus sprouts shot out, discharging an unhealthy, vitiated and fetid matter, and bleeding at the slightest touch. Her general health rapidly failed, and when she became an inmate of the Hospital, she was reduced to the extreme of emaciation and debility from the hemorrhage exhausting discharges, want of food and care. She improved under the administration of tonic medicines and proper food, so that at the end of ten days from her admission I felt warranted in the attempt to save her life by operative means. As the base of the tumor seemed to be limited to the exterior and lower portion of the humerus, and the shaft of the bone seemed sound above the middle, it was thought best [also in view of her debilitated condition,] to amputate at the insertion of the pectoral and latissimus dorsi muscles, which was accordingly carried into effect on the 15th of April, at which operation, in conjunction with other medical friends, the Editor of this Journal, kindly assisted. Union of the flaps by first intention ensued, and at the end of two weeks only a small portion of the wound remained uncicatrized. At this time her health had vastly improved; her appetite was good and her spirits buoyant, and she was indulging in hopes of returning to her home. After a sleepless and painful night, at my morning visit she called my attention to the unhealed portion of the wound; it was about the size of a dime; instead of its healthy, granulated surface, it presented a glassy surface, adematous, and exuding a viscous and fœtid secretion. She complained of deep-seated, lancinating pain; daily this enlarged, involving the adjoining integuments until it was obvious that a renewal of the disease had taken place.

A few days after she was seized with a severe chill; her appetite failed and she urgently implored for another operation; preferring the danger of death from the attempts, to the anguish of another fungous tumor. I was very much disappointed in the unfavorable termination; for from the character of the bony tissue at the place where it was divided by the saw in the amputation, all of us who examined it, were quite confident that it would not reappear.

I ought to have mentioned above, that through the kindness of Dr. John Boardman, my colleague, I was furnished with the microscopic appearance of the excised mass. He states that the tumor seemed to be composed of fibrous tissue, filled with cells of a large size, most of which were of an oval irregular shape. The cells were filled with a granular matter; did not find any distinct nucleus, but each cell contained several large granules. Free oil globules, blood corpuscles and pus cells, were also found.

Upon consultation, it was deemed advisable before further impairment of her general health, to remove the stump at the shoulder joint; this was accordingly done on the 15th, of the month; she bore the operation well; in two days she entirely rallied from its effects and has been daily improving in strength and health. The wound has almost entirely united by primary adhesion; most of the ligatures have come away and the small central portion, as yet uncicatrized, looks healthy and is daily contracting. It is to be hoped that her fortitude and courage will be rewarded by entire restoration to health.

In designating the tumor, I have followed the nomenclature of John Erichsen.

Buffalo, 25th May, 1862.

